# Ultrafast and Resist-Free Nanopatterning of 2D Materials by Femtosecond Laser Irradiation

**DOI:** 10.1021/acsnano.2c09501

**Published:** 2023-04-19

**Authors:** Alessandro Enrico, Oliver Hartwig, Nikolas Dominik, Arne Quellmalz, Kristinn B. Gylfason, Georg S. Duesberg, Frank Niklaus, Göran Stemme

**Affiliations:** † Division of Micro and Nanosystems, KTH Royal Institute of Technology, Malvinas väg 10, 10044 Stockholm, Sweden; ‡ Institute of Physics, EIT 2, Faculty of Electrical Engineering and Information Technology, University of the Bundeswehr Munich & SENS Research Center, Werner-Heisenberg-Weg 39, 85577 Neubiberg, Germany

**Keywords:** direct writing, photoablation, graphene, MoS_2_, PtSe_2_, two-photon patterning

## Abstract

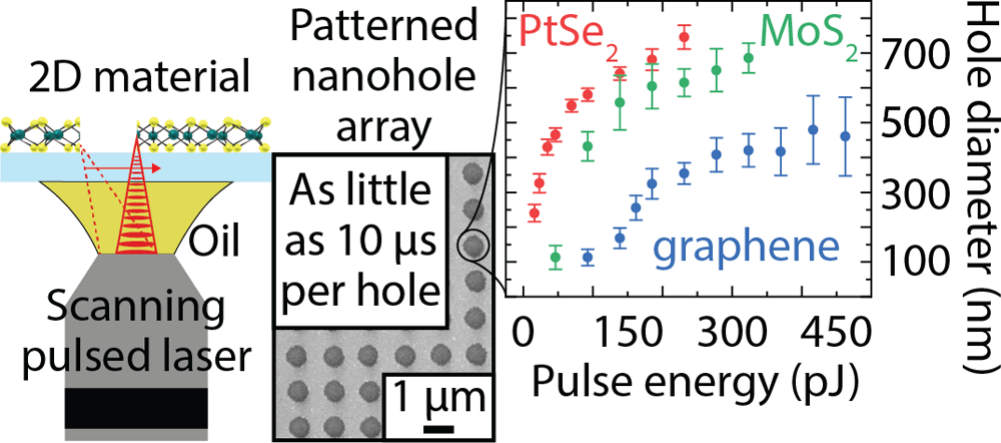

The performance of two-dimensional (2D) materials is promising for electronic, photonic, and sensing devices since they possess large surface-to-volume ratios, high mechanical strength, and broadband light sensitivity. While significant advances have been made in synthesizing and transferring 2D materials onto different substrates, there is still the need for scalable patterning of 2D materials with nanoscale precision. Conventional lithography methods require protective layers such as resist or metals that can contaminate or degrade the 2D materials and deteriorate the final device performance. Current resist-free patterning methods are limited in throughput and typically require custom-made equipment. To address these limitations, we demonstrate the noncontact and resist-free patterning of platinum diselenide (PtSe_2_), molybdenum disulfide (MoS_2_), and graphene layers with nanoscale precision at high processing speed while preserving the integrity of the surrounding material. We use a commercial, off-the-shelf two-photon 3D printer to directly write patterns in the 2D materials with features down to 100 nm at a maximum writing speed of 50 mm/s. We successfully remove a continuous film of 2D material from a 200 μm × 200 μm substrate area in less than 3 s. Since two-photon 3D printers are becoming increasingly available in research laboratories and industrial facilities, we expect this method to enable fast prototyping of devices based on 2D materials across various research areas.

Two-dimensional (2D) materials are atomically thin films with physical, chemical, and mechanical properties that differ from the bulk due to quantum confinement, high surface-to-volume ratios, or surface charge.^1^ The thinnest 2D materials are composed of a single layer of atoms, while other 2D materials are composed of multiple atomic layers.^2−4^ Graphene and hexagonal boron nitride (2D-hBN) belong to the first class of 2D materials, while the latter class includes the transition metal dichalcogenides (TMDs), such as molybdenum disulfide (MoS_2_) and platinum diselenide (PtSe_2_). The properties of 2D materials depend on the number of unit layers and the surrounding substrate material.^4,5^ For instance, the electrical bandgap of semiconducting 2D materials can change with the number of layers, which is interesting for device applications.^6^ The resulting electrical, mechanical, and optical properties of 2D materials are interesting for transistors, photodetectors, chemical and pressure sensors, as well as for light emitting devices, energy conversion, and storage.^7−11^ Large-area synthesis and transfer methods can provide 2D materials with high quality on various types of substrates.^12^ However, the fabrication of devices requires structuring the continuous 2D material layers, which often degrades the material properties due to contamination or damage. For example, optical lithography and laser interference lithography are widely available processes for structuring 2D materials, but they require coating the 2D material with a protective resist mask during etching. The process steps of coating, development, and removal of the photoresist after patterning can damage the 2D material and cause polymeric residues, which degrades the layer quality.^13,14^ Additionally, the resolution of optical lithography is limited by diffraction, which constrains the minimum feature size. Resist-based electron beam lithography and extreme UV stepper lithography can achieve features below 100 nm but still risk contaminating the 2D material with resist residues. Patterning by subtractive direct writing with electron beam systems has achieved structures with high resolution without contaminating the 2D material by resist residues.^15^ However, direct writing using electron or focused ion beams (FIB) requires a long processing time that scales with the writing area. Direct writing using continuous or long-pulsed lasers has been reported for patterning 2D materials, laser-induced carbonization or graphitization of different materials, or reduction of graphene oxide with diffraction-limited resolution.^16−20^ Laser ablation is faster than electron or FIB direct writing, but conventional lasers are limited to relatively large features ranging from millimeter to single-digit micrometer line width. In contracts, short-pulsed lasers can achieve submicrometer patterning of 2D materials.^21−27^ Most reports describe setups with a fixed laser beam and a mechanical scanning system that moves a piezoelectric stage to expose the desired area of the sample. This configuration strongly limits the patterning speed and the maximum area of exposure.

To address these bottlenecks, we present the ultrafast and resist-free nanopatterning of 2D materials using a commercial two-photon 3D printer with a beam scanning system (Figure 1a). Direct writing does not require coating the sample with a masking layer, thereby avoiding contamination. We patterned graphene, MoS_2_, and PtSe_2_, reaching a subwavelength resolution (≥100 nm hole diameter) at high throughput (∼3 s to clear a 200 μm × 200 μm area). The continuous layers were patterned without damaging the substrate or the surrounding material. Since two-photon 3D printers are becoming widely available in research laboratories and industrial manufacturing, we anticipate a widespread interest in this facile and easy-to-automate approach for fast prototyping of devices based on 2D materials.

**Figure 1:**
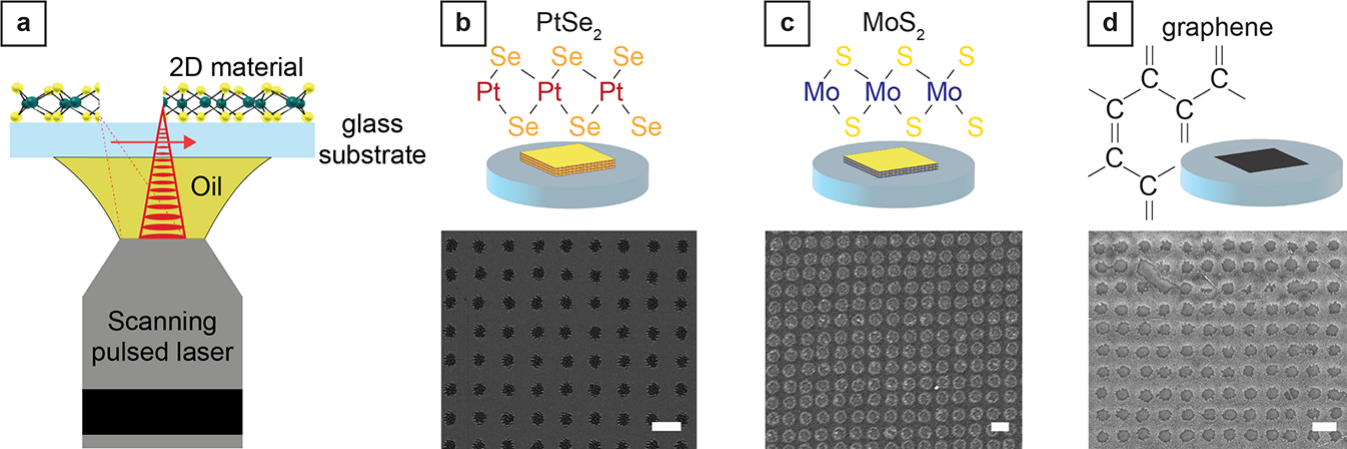
Nanopatterning of 2D materials using a two-photon 3D printer. (a) 3D schematic of the laser writing approach. The 2D material is either grown or transferred on the front side of a thin glass coverslip. Patterning of the 2D material is performed using a 63× objective (NA = 1.4) with immersion oil contacting the backside of the coverslip side. (b), (c), and (d) present the three 2D materials patterned with this method, platinum diselenide (PtSe_2_), molybdenum disulfide (MoS_2_), and graphene, respectively. The lower row displays nanohole arrays in the different 2D materials with decreasing hole-to-hole pitch. Scale bar, 1 μm for PtSe_2_, 500 nm for MoS_2_, and 300 nm for graphene.

## Results and Discussion

We demonstrate the possibility of using a commercially available two-photon 3D printer to achieve ultrafast resist-free nanopatterning in 2D materials of general interest: PtSe_2_, MoS_2_, and graphene. Multilayer PtSe_2_ films were grown on borosilicate glass coverslips, while monolayer graphene and multilayer MoS_2_ films were transferred onto the glass coverslips. The continuous films of 2D materials were then patterned by femtosecond laser exposure (780 nm wavelength, 80–120 fs pulse duration, 80 MHz repetition rate), generating nanohole arrays in the three materials (Figure 1b–d). For the exposure, a two-photon 3D printer (Photonic Professional GT2, Nanoscribe, Germany) was used in oil immersion configuration with a 63× objective (see Methods section for details). The 2D materials were patterned using a transmission configuration (Figure 1a). Immersion oil is applied between the backside of the thin glass coverslip and the objective, while the 2D material is placed on the front side. The laser beam is transmitted through the substrate and illuminates the 2D material on the front surface. The oil matches the refractive index of the lens and the substrate, increasing the maximum patterning resolution. Working in transmission mode avoids any contamination of the 2D material since there is no physical contact between the 2D material and the immersion oil. This configuration requires the substrate material to be transparent at the wavelength of the laser (780 nm). Additionally, the maximum physical thickness of the substrate that can be used is limited by the working distance of the objective (when the upper surface of the standard 170 μm thick glass coverslip is in focus, the free working distance between the bottom surface and the used objective is 190 μm).

**Figure 2:**
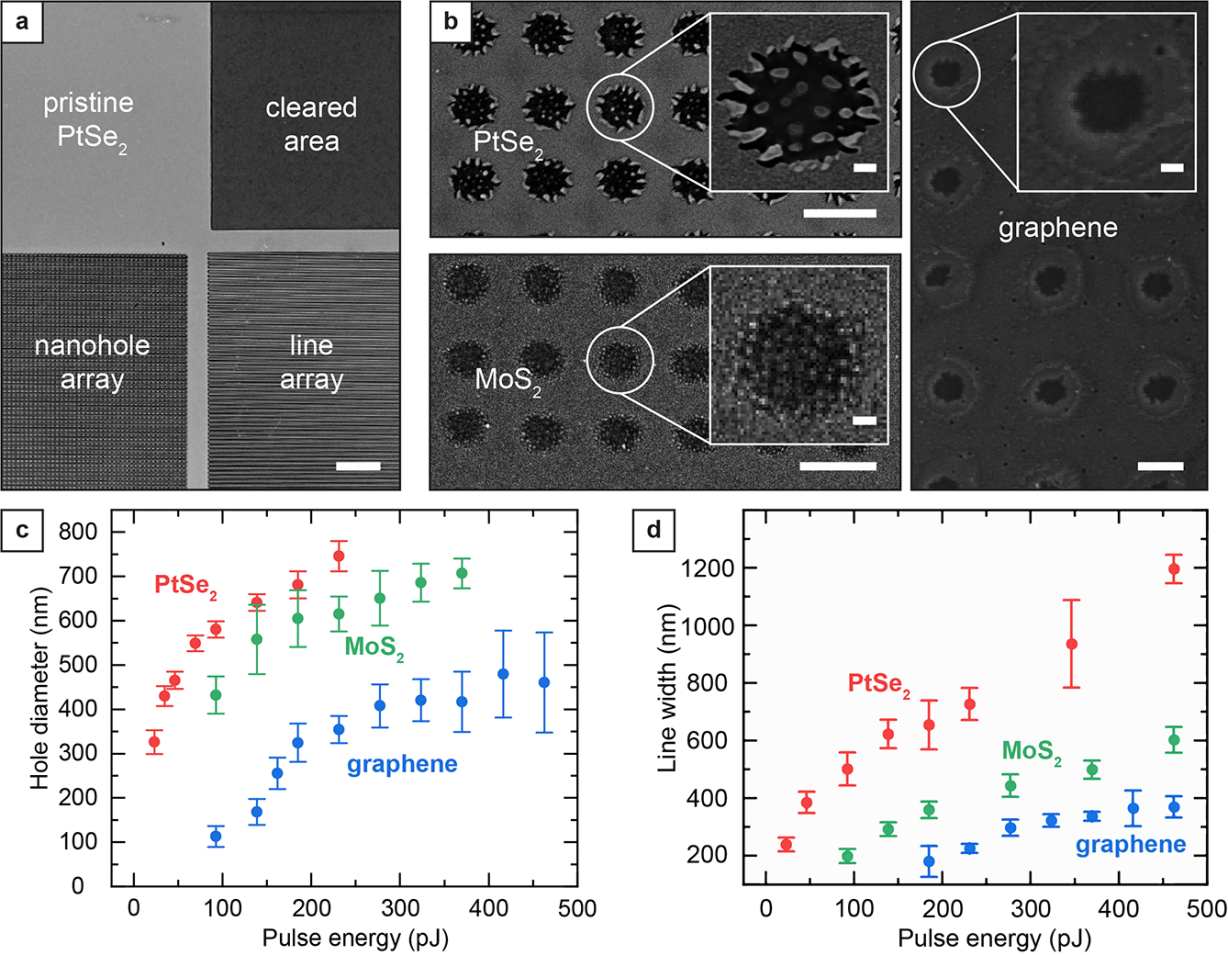
Laser patterning modes and study of maximum pattern resolution and processing speed. (a) Optical image showing a PtSe_2_ film in its pristine form (top left), laser patterned with a nanohole array (bottom left) and line array (bottom right), as well as an area where the PtSe_2_ film was completely removed by laser patterning (top right). Scale bar, 10 μm. (b) Zoom-in SEM images of the patterned holes with single hole inset. Scale bar, 1 μm and 100 nm (inset) for PtSe_2_ and MoS_2_, 200 nm and 50 nm (inset) for graphene. (c) Average diameter of the nanoholes patterned in the different 2D materials as a function of the laser pulse energy. Each data point and associated error bars correspond to the average and the standard deviation of at least 20 measurements on distinct holes. (d) Average width of individual lines patterned in the different 2D materials as a function of the laser pulse energy. Each data point and associated error bars correspond to the average and the standard deviation of at least 20 measurements on distinct lines.

To characterize the patterning capabilities of this approach, we patterned an array of nanoholes, lines, and cleared rectangular areas in the 2D materials. Nanohole arrays enable important applications in optical sensing, plasmonics, and electrochemistry, while lines can outline the boundaries of the active areas in functional devices.^28−31^ Removing the 2D material surrounding the structure of interest is beneficial to ensure electrical insulation between the two portions of 2D material and when transferring the 2D material after patterning.^32^ We found the highest resolution of 100 nm for individual holes, a maximum effective speed of 50 mm/s when patterning line arrays, and the possibility to clear a 200 μm × 200 μm substrate area from the 2D material within in less than 3 s of processing (Figure 2a). PtSe_2_, MoS_2,_ and graphene could all be patterned using the available pulse energy range (up to 550 pJ) and the lowest exposure time allowed by the instrument control software (10 μs per individual spot).

During illumination, the 2D material under laser exposure can undergo several reactions due to thermal and nonthermal effects (plasma conversion, melting and resolidification, sublimation, conversion to volatile compounds, and ballistic removal due to accelerated atoms produced during plasma).^22,25,33,34^ During the high fluence exposure, the laser energy transmits through the transparent glass coverslip with minimal absorption. Most of the absorption occurs on the top surface of the coverslip in the 2D material. A combination of sublimation and plasma formation results in material removal at the center of the laser spot where the pulse fluence is high. Melting and oxidation can occur at the edges of the illuminated area where the pulse fluence is lower than the pulse center. The removed material is dispersed in the surrounding environment and can potentially deposit on the device substrate. However, the minimal 2D material thickness results in negligible sample contamination (not enough to be qualitatively or quantitatively characterized). By characterizing the morphology of laser patterned holes in PtSe_2_ and MoS_2_, we can observe residues at the edge and inside the illuminated region (Figure 2b, see Figure S1 in the Supporting Information). The residues are likely Pt- and Mo-rich islands (metallic Pt atoms or substoichiometric PtSe_*x*_ residues for PtSe_2_ and metallic or oxidized Mo atoms for MoS_2_), but they are present and connect to the films only near the holes (100 nm or less). In the case of graphene, the SEM and AFM analyses did not reveal any residues at the edge or inside the illuminated area. However, AFM profiling detected a buckling of the graphene layer around the holes (within 100 nm or less from the holes), which we attribute to the localized heating during exposure that produces a plastic deformation in the film where the fluence is insufficient for laser ablation.^35^ For low pulse energy (<250 pJ, Figure S1), there is only a slight elevation of the film surrounding the holes with respect to the rest of the monolayer. When higher pulse energies are used, folding of the monolayer occurs, changing the shape of the hole from a circular to a polygon-like shape (with straight edges where folding occurs, see Figure S2 in Supporting Information).^36^

To evaluate the range of achievable feature sizes, we generated nanohole arrays with different pulse energies while keeping the exposure time constant, imaged the resulting nanohole array with SEM, and measured the average hole diameter (Figure 2c, see details in the Methods section). To generate nanohole arrays in the 2D materials, each hole position was irradiated for 10 μs (corresponding to 800 pulses with the 80 MHz repetition rate of the system) since this exposure value was close to the lower limit available in our 3D laser printing tool. We observed a significantly higher susceptibility to laser exposure for PtSe_2_ multilayer films than for the other two materials. Pulse energies as low as 20 pJ resulted in reliable patterning of PtSe_2_. Multilayer MoS_2_ displayed a similar increase in hole diameter with increasing pulse energy. For the same pulse energy, the average hole diameters in MoS_2_ films were larger than in monolayer graphene, suggesting a dependency on the number of layers and the optical properties of the 2D material. We achieved the highest resolution in monolayer graphene with hole diameters down to 100 nm at a pulse energy of 90 pJ. Increasing pulse energies enlarged the hole diameter up to 400 nm. We found the same trend for the energy threshold when patterning line arrays. (Figure 2d). We believe these differences are related to the binding energies of the atoms composing the three 2D materials. The formation energy of selenium vacancies in PtSe_2_ is calculated to be around 1.5 eV, at least 4 times lower than the formation energy of sulfur vacancies in MoS_2_ (around 5.8 eV) or vacancy formation in graphene (around 7.4 eV).^37−39^ The differences in the formation energies for the vacancies reported in the literature are coherent with the different energy thresholds observed for hole formation in the different 2D materials (Figure 2c). Since the size of the holes is influenced by the presence of residues for PtSe_2_ and MosS_2_ films and by folding for graphene monolayers, simple models for single-pulse laser ablation cannot accurately relate the pulse energy with the resulting hole size (see Figure S3 in the Supporting Information).^40^

While laser direct writing has been previously used for submicrometer patterning of 2D materials with different short-pulsed laser systems, there are issues concerning the consistency of the feature size due to the manual focusing and the processing throughput is limited for nonbeam-scanning systems.^21−27^ We circumvented these limitations by using the built-in interface finder of the 3D printer system to achieve automatic high-resolution focusing (±1 μm on the *z*-axis) and the nominal scanning speed of up to 2 × 10^5^ μm/s to clear 200 μm × 200 μm areas from 2D material in 3 s by patterning an array of lines with 1 μm spacing using a pulse energy of 500 pJ (see Figure S4 in the Supporting Information). By decreasing the pulse energy or increasing the line separation, we could pattern an array of individual lines instead of clearing entire areas. Alternatively, we could create a nanohole array pattern in the 2D material in as little as 6 μs per hole/position. This minimum exposure value corresponds to 480 pulses per individual position, suggesting that further lowering exposure time could still be compatible with 2D material patterning. Practically, the throughput is limited by the idle time that the hardware requires to scan the laser beam from one position to the following one and stabilize the position of the laser beam (down to 150 μs).

**Figure 3:**
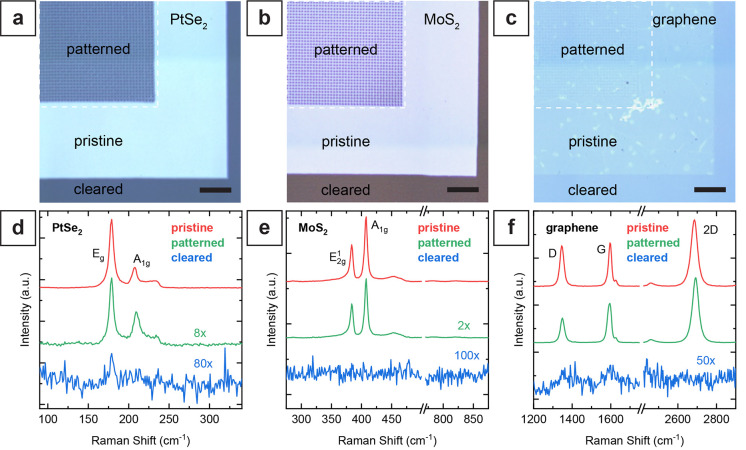
Raman characterization of the patterned 2D materials on glass coverslips. (a–c) Optical microscope images of patterned PtSe_2_, MoS_2_, and graphene samples. Raman scans were performed on pristine, patterned, and cleared regions for each material. Scale bars, 10 μm. (d–f) Averaged Raman spectra of pristine (red), patterned (green), and cleared (blue) regions of PtSe_2_, MoS_2_, and graphene. The spectra of the patterned areas show the expected modes with reduced intensity due to the material ablation. The mode characteristics of each material show no significant degradation of material quality for the patterned regions. The area where the 2D material was fully removed only shows weak or no corresponding Raman signal (blue line). In (d), (e), and (f), the spectra with low intensity are presented with different scaling factors for better visibility: 8× for patterned PtSe_2_, 80× for areas cleared from PtSe_2_, 2× for patterned MoS_2_, 100× for areas cleared from MoS_2_, and 50× for areas cleared from graphene.

To analyze the effect of the 2D material patterning by laser direct writing on the surrounding (remaining) 2D material, we used Raman spectroscopy and X-ray photoelectron spectroscopy (XPS) (see details in the Methods Section and Figure S5 in the Supporting Information for the description of the evaluated pattern). We selected different combinations of pitch and pulse energy to investigate the potential degradation of the material surrounding the exposed areas. Specifically, we patterned nanohole arrays with large hole-to-hole pitches (1 and 4 μm) using high-energy pulses (90 pJ for PtSe_2_, 190 pJ for MoS_2_, and 500 pJ for graphene) to observe potential long-range thermal effects. By using a combination of small pitch (300 nm) and low-energy pulses (185 pJ or lower), we could also obtain smaller holes and a higher hole density of the array, maximizing the fraction of the 2D material close to the exposed sites and get further insight into the quality of the remaining material after laser exposure. We performed the Raman analysis for the three 2D materials by recording Raman spectra of pristine material regions, regions with patterned nanohole arrays, and regions where the 2D material film was removed entirely (Figure 3a–c, see Figure S6 in the Supporting Information for the detailed analyses of the films directly after growth or wet transfer). The Raman spectra were obtained from area scans (8 × 8 μm^2^ area for PtSe_2_ and MoS^_2_^ and 40 × 40 μm^2^ for graphene) on the pristine, patterned, and cleared regions of the films. Each area scan consists of multiple spectra obtained by scanning the laser spot over the area (details in the Methods section, and the additional discussion in the Supporting Information).

The averaged spectrum of the patterned PtSe_2_ region resembles the spectrum of the pristine film (Figure 3d). The Raman spectrum features two characteristic modes E_g_ and A_1g_, corresponding to the in-plane and out-of-plane atomic vibrational motion, respectively.^28^ The E_g_ and the A_1g_ modes in the pristine PtSe_2_ region are observed at 178.8 and 206.9 cm^–1^, respectively, in line with the literature.^41−46^ In addition, a third peak at ∼233 cm^–1^ of lower intensity was attributed to the overlapping longitudinal optical (LO) modes A_2u_ and E_u_.^41^ While the intensities of the E_g_ and A_1g_ modes are generally of similar magnitude in bulk PtSe_2_, the intensity of the observed A_1g_ mode of the grown PtSe_2_ films was 3 times lower than the E_g_ mode intensity, confirming the few-layer nature of the PtSe_2_ film.^41^ The E_g_ mode intensity of the patterned PtSe_2_ spectrum reduces to ∼11% of the pristine spectrum. This reduction is attributed to the removal of material at the hole positions. Nevertheless, since both characteristic modes are present and there is no significant increase in the line width, we conclude that the integrity of the remaining PtSe_2_ is preserved. On the contrary, no clear fingerprint of PtSe_2_ can be observed in the spectrum of the cleared region. Only a small peak of the E_g_ mode with an intensity of only 0.5% of the pristine E_g_ mode intensity remains.

Similar to the case of PtSe_2_, the averaged spectrum of the patterned MoS_2_ region resembles the spectrum of the pristine film (Figure 3e). In the case of MoS_2_, the Raman spectrum of the unpatterned transferred films features the E^1^_2g_ and A_1g_ mode peaks, respectively, at 383.5 and 407.4 cm^–1^, in line with previously reported values.^12,47−52^ The averaged spectrum obtained from the patterned region resembles the pristine spectrum with a decrease in intensity to 45% of the pristine spectrum. Without noteworthy differences in the spectrum, the reduction in intensity is mainly attributed to the removal of MoS_2_. Additionally, the absence of a peak around 823 cm^–1^, where a Mo=O stretching mode would be expected for oxidized molybdenum (MoO_3_), also indicates negligible oxidation of the MoS_2_.^53,54^ The analysis of the mode difference A_1g_ – E^1^_2g_, a function of the number of atomic layers in the MoS_2_ film,^50^ allows access to the film thickness. In both pristine and patterned spectra, the mode difference A_1g_ – E^1^_2g_ calculates to 24 cm^–1^, confirming the few-layer nature of the MoS_2_.^50,51^ The spectrum of the cleared region does not show any remaining peaks but only noise of the CCD detector, confirming effective ablation of the MoS_2_.

Lastly, we observed no significant spectral difference when comparing pristine and patterned graphene areas (Figure 3f). The Raman analysis of graphene yielded the characteristic modes (2D and G bands) at 2686 and 1595 cm^–1^, respectively, in line with previous reports.^12,55−58^ The large peak intensity ratio (*I*_2D_/*I*_G_) of approximately 4 indicates the monolayer nature of the graphene film.^56^ Moreover, the narrow fwhm of the 2D band of 33 cm^–1^ is comparable to reported values for monolayer graphene.^12,57^ The analysis of the pristine graphene region yields intensities of the defect-activated D band (located at 1349 cm^–1^) ranging from less than 10% of the G band (*I*_G_/*I*_D_ < 10%) to similar intensities (*I*_G_/*I*_D_ ∼ 1). These results indicate variability in the quality of pristine materials (Figure S6).^58^ Pristine and patterned graphene regions feature similar Raman spectra without significantly increasing D peak intensity. Moreover, the overall peak intensity is almost identical, which we attribute to a smaller hole size obtained in graphene than in MoS_2_ and PtSe_2_ (Figure 2c). In the cleared region, the narrow 2D, G, and D bands are absent, and the only significant yet weak signal comes from the broad D and G bands of amorphous carbon at 1350 and 1600 cm^–1^.^59^ Moreover, high-resolution Raman mapping confirms that the film surrounding the laser patterned holes is uniform and resembles pristine 2D material films (see Supporting Information for description of the experiment and Figure S7).

**Figure 4:**
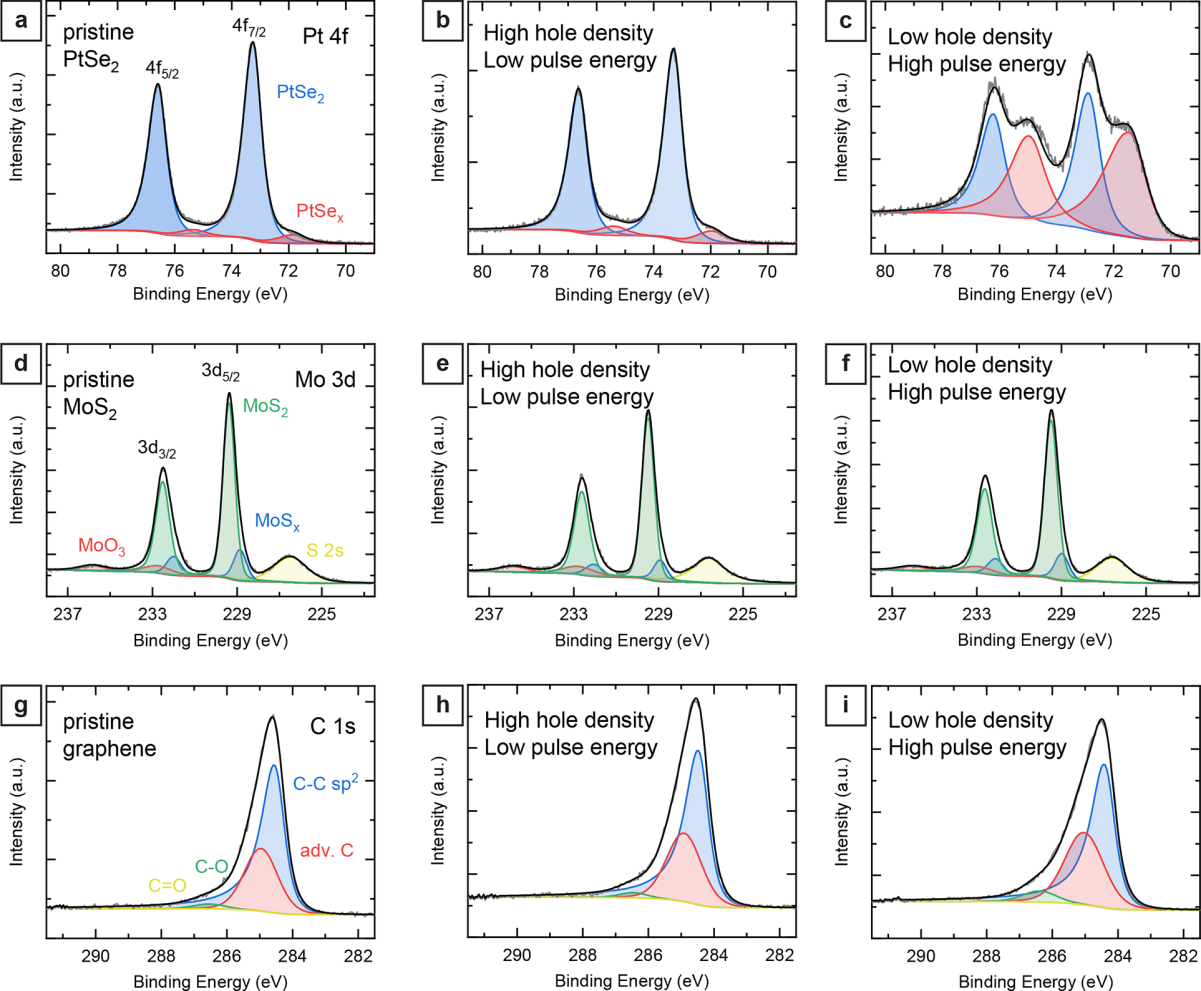
Comparison between pristine and laser structured 2D materials by X-ray photoelectron spectroscopy (XPS). (a) Characteristic Pt 4f spectrum of pristine PtSe_2_ with the Pt 4f doublet binding energies at 73.3 eV for 4f_7/2_ and 76.6 eV for 4f_5/2_. (b) Pt 4f orbital of PtSe_2_ film exposed using a high-density nanohole pattern (300 nm hole-to-hole pitch) and using low pulse energy (20 pJ). (c) Pt 4f orbital of PtSe_2_ film exposed using a low-density nanohole array (1 μm hole-to-hole pitch) and high pulse energy (500 pJ). (d) Characteristic Mo 3d spectrum of the transferred MoS_2_ film with the Mo 3d doublet binding energies at 229.4 eV for 3d_5/2_ and 232.5 eV for 3d_3/2_. (e) Mo 3d orbital of MoS_2_ film exposed using a high-density nanohole pattern (300 nm hole-to-hole pitch) and using low pulse energy (100 pJ). (f) Mo 3d orbital of MoS_2_ film exposed using a low-density nanohole array (4 μm hole-to-hole pitch) and high pulse energy (500 pJ). (g) Carbon 1s spectrum of the pristine graphene layer with the asymmetric C–C sp^2^ band binding energy at 284.5 eV. (h) Carbon 1s orbital of graphene exposed using a high-density nanohole pattern (300 nm hole-to-hole pitch) and using low pulse energy (150 pJ). (i) C 1s orbital of graphene exposed using a low-density nanohole array (4 μm hole-to-hole pitch) and high pulse energy (500 pJ).

To analyze potential chemical changes in the 2D materials, XPS measurements were performed on patterned regions. Additionally, reference measurements were performed on pristine regions of the films. To compare the XPS spectra of pristine films and nanohole arrays with high and low density, we analyzed the Pt 4f orbitals of platinum, the Mo 3d orbitals of molybdenum, and the C 1s orbitals of carbon (Figure 4, see Figure S8 in the Supporting Information for the XPS spectra of selenium (Se 3d orbital) and sulfur (S 2p orbital)). The XPS spectrum for pristine PtSe_2_ films shows the characteristic Pt 4f doublet binding energies at 73.3 eV for 4f_7/2_ and 76.6 eV for 4f_5/2_ (Figure 4a).^42,44^ The signal was fitted by two Pt 4f doublets, one major contribution corresponding to Pt(IV) atoms bound to two selenium atoms and a small percentage of substoichiometric bound Pt atoms (shifted to lower binding energies by ∼1.2 eV), which indicates selenium vacancies at the surface and edges of the PtSe_2_ nanocrystals. The Pt 4f orbital of the patterned PtSe_2_ area using a low pulse energy and high hole density (20 pJ, 0.3 μm spacing) shows negligible differences compared to pristine PtSe_2_ (Figure 4b).^44^ Only a minimal increase of the substoichiometric bound Pt atoms is visible, which we attributed to Se vacancies at the increased edge area of the hole structure. In contrast, we found more significant differences when comparing pristine and patterned PtSe_2_ films with high-energy pulses and low hole density (500 pJ, 1 μm spacing). The XPS spectrum showed a substantial increase in the substoichiometric bound Pt atoms (Figure 4c). Additionally, we observed a shift of the PtSe_*x*_ component toward lower binding energies, suggesting a significant loss of selenium atoms due to partial thermal degradation of the PtSe_2_ film near the patterned holes.

For the pristine MoS_2_ films, we found the Mo 3d doublet binding energies at 229.4 eV for 3d_5/2_ and at 232.5 eV for the 3d_3/2_ band. The sulfur S 2s band was observed at 226.5 eV (Figure 4d). The observed MoS_2_ and sulfur band energies agree with previous reports.^42,60−63^ The Mo 3d spectra were fitted using two doublets for the MoS_2_ bands. Like the PtSe_2_ spectra, the fitted spectra consist of a major contribution for Mo (IV) atoms bound to two sulfur atoms and a minor MoS_*x*_ contribution at a lower binding energy of 228.9 eV for 3d_5/2_, corresponding to sulfur vacancies.^62,63^ In addition, we observed another doublet with the Mo 3d_5/2_ band at 232.8 eV, corresponding to Mo(VI) of MoO_3_, indicating a low degree of oxidation (8% MoO_3_) of the pristine MoS_2_ film.^42,60−63^ Unlike patterned PtSe_2_ films, we did not observe significant differences when comparing pristine MoS_2_ films and nanohole-structured areas, regardless of the pulse energy. These results confirm that this laser direct writing approach preserves the integrity and property of the MoS_2_ film adjacent to the patterned structures (Figure 4e,f).

For pristine graphene monolayers, the XPS spectrum shows the carbon 1s orbital with the asymmetric peak and a high-energy tail, as expected for a graphene layer (Figure 4g).^55,64,65^ The asymmetric peak results mainly from the C=C sp^2^ bonds of graphene at an energy of 284.5 eV and, to a lesser extent, from adventitious sp^3^ carbon with band energy centered around 285.0 eV.^55,64,66−68^ Additionally, a small contribution of the oxidized carbon groups C–O (hydroxyl) is observed at 286.5 eV with ∼3% of the C=C sp^2^ band intensity. The applied curve fits also included a band for the C=O groups (carbonyl) at 287.5 eV; however, no significant contribution was observed.^55,64,66−68^ The surface contamination with adventitious carbon results in the intense signal contributions of sp^3^ carbon and C–O bonds. After patterning using low-energy laser pulses, no significant changes were observed in the spectrum. The relative intensity of the C=C sp^2^ peak compared to the sp^3^ carbon is decreased slightly from 2.3 to 2.1 with respect to pristine films, which we attribute to the reduced total area (Figure 4h). Also, the increased adventitious carbon on the patterned areas and minor degradation of the sp^2^ network near the holes might account for the reduced relative sp^2^ signal. For high-energy pulses, the intensity of the high-energy tail of the spectrum expanded further (Figure 4i). The relative intensity of the C–O peak compared to C=C sp^2^ band intensity increased from around 3% to 8%. We attribute this increase to the oxidation of the graphene layer around the patterned holes.

Based on our observations, removing material is a complex phenomenon that depends on the illuminated material and the pulse energy and deserves further attention in future studies. In the case of dichalcogenides, we believe sulfur and selenium are the predominantly removed elements. While platinum and molybdenum are partially carried away by the sublimating material or the ejected plasma during the laser illumination, the remaining material resolidifies either at the border of the illuminated areas or as isolated islands inside the formed holes. To get information regarding these residues, we have run Raman and XPS measurements on areas cleared from the 2D material film (see Figure S9 in the Supporting Information for the XPS spectra). Both analyses revealed a low intensity signal of platinum and molybdenum. In the case of the MoS_2_ films, we observed MoO_*x*_ residues, indicated by the observed Mo 3d doublet shifted to a higher binding energy than the pristine MoS_2_ spectrum. These oxides residues are presumably amorphous and low in quantity, as no MoO_3_ Raman mode was observed (Figure 3). In the case of PtSe_2_, a mixture of metallic Pt and substoichiometric PtSe_*x*_ is observed with traces of selenium bound to the Pt as PtSe_x_.

**Figure 5:**
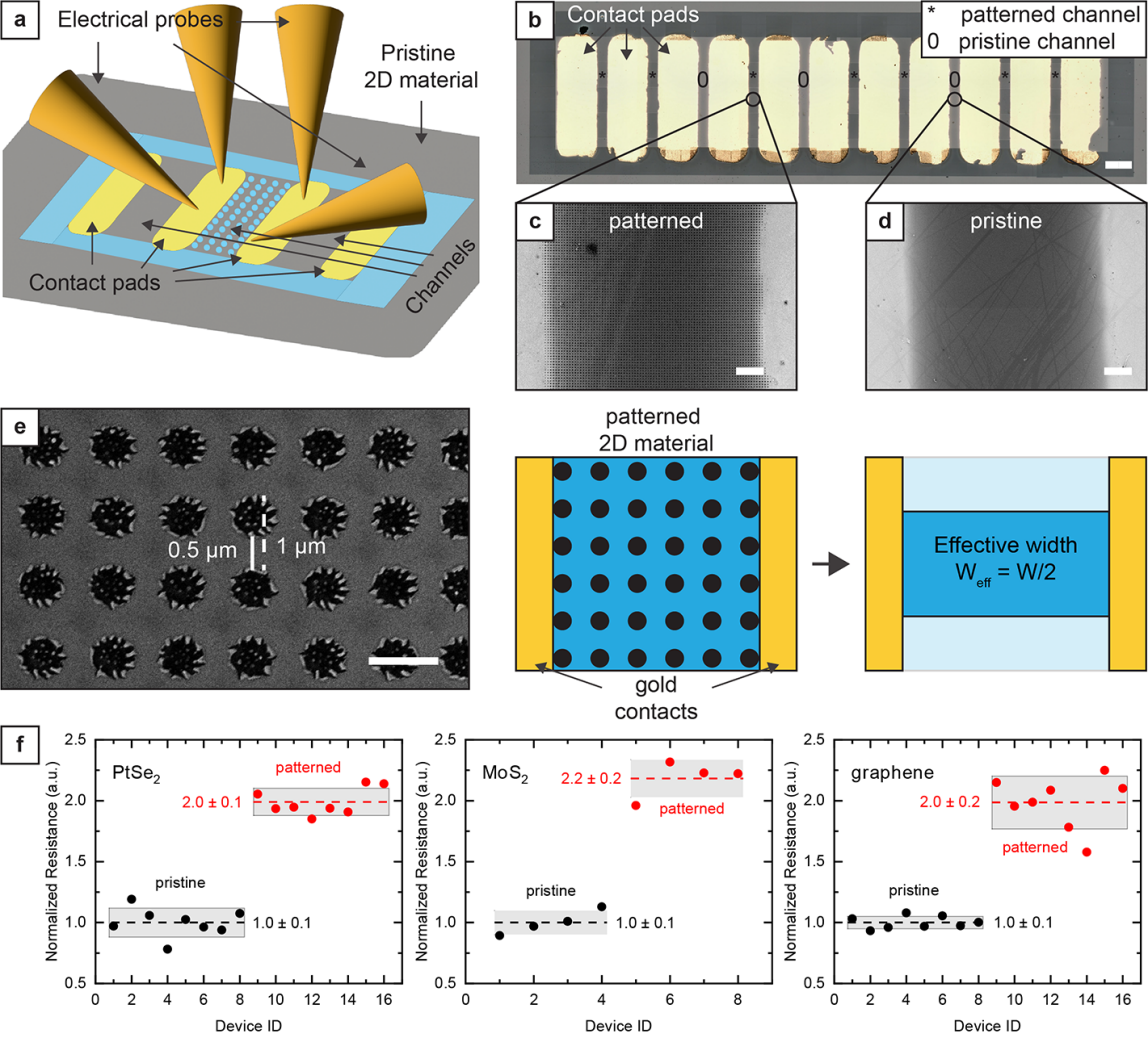
Electrical characterization of 2D materials patterned by high-speed laser direct writing. (a) Schematic of the device configuration for the electrical characterization of the patterned 2D materials. The 2D material surrounding the electrode array was removed by laser direct writing to electrically isolate the array of devices from the surrounding 2D material layer and define the device channel geometry. Depending on the resistance value, either a two- or four-point probe measurement of the electrical resistance was performed by sweeping either the current or the voltage. (b) Optical microscope image of 10 test structures for electrical characterization of the 2D material channels. Each test structure consists of two gold contact pads and a channel with either a pristine (marked with “0”) or a patterned (marked with “*”) 2D material patch connecting the two electrodes. Scale bar, 200 μm. (c) and (d) are SEM images showing sections of, respectively, patterned and pristine channels between the gold contact pads. Scale bar, 10 μm. (e) SEM image and schematic illustrating the physical constriction of the conductive path due to the patterned nanohole array. Since the hole diameter and pitch are 500 nm and 1 μm, respectively, the effective width of the channel due to the nanohole array is halved. Scale bar, 1 μm. (f) Measured resistances of pristine channels and channels with nanohole array patterns normalized on the average resistance of the pristine channels. The channels consisting of the patterned 2D materials have a significantly higher resistance (2 times higher) than those consisting of a continuous 2D material film due to the constriction of the conductive path.

While we have no evidence of significant material degradation from our Raman and XPS characterizations, we run a simple electrical characterization of the pattern films to investigate possible edge effects or material degradation on the film conductivity (Figure 5a). The grown or transferred 2D materials were structured by laser direct writing. Metal contacts were deposited by shadow masking, thus avoiding any contact with resists and solvents. To assess the effect of the nanohole pattern on the electrical resistance of the films, we deposited an array of electrical contacts using gold evaporation through a stencil designed to leave 100 μm wide channels of 2D materials between each contact pair. We selectively patterned half of the 2D material channels using a nanohole pattern (Figure 5b,c). We tuned the laser power to obtain 500 nm wide holes and used 1 μm spacing between the center of first-neighboring holes. After laser patterning with the parameters described in the previous section, we characterized the resulting nanohole array by SEM, observing that we formed 500 nm wide holes, resulting in a reduction of the effective width at the constriction by a factor of 2 (Figure 5d,e). We then characterized the samples by direct current (DC) measurements. The devices displayed a linear resistive behavior, and the value of resistance for patterned channels was higher than the one of continuous channels by a factor of 2 for all materials, in line with our expectation based on the decrease of effective width (Figure 5f, see also Figure S10 in the Supporting Information for the *I*–*V* curves and the absolute value of channel resistance of the different samples and materials). Our data set shows no significant change in film conductivity due to edge effect or material degradation. Moreover, the residues inside the PtSe_2_ and MoS_2_ holes do not seem to contribute to the conduction and are instead isolated islands of materials. While a complete understanding of the carrier transport in the patterned 2D films is difficult to obtain, it would be interesting to investigate the exact dynamics in future works and use more advanced structures, such as field effect transistors.

As previously described, the presented patterning configuration requires a thin substrate transparent to the laser light, which constitutes a limitation of the method we demonstrated in this work. However, a configuration for direct laser exposure of the 2D material on the substrate surface is also feasible for 2D material patterning. Using a suitable high-magnification air-immersion objective, we believe it would be possible to pattern 2D materials on opaque substrates such as silicon, albeit with somewhat lower resolution than with the oil immersion configuration presented in this work. However, the selection of possible substrate materials would still be limited to materials not experiencing ablation at similar laser irradiation conditions as the one used for patterning the 2D material. Further studies will be necessary to investigate the capabilities and limitations of such an approach on different substrate materials.

## Conclusions

In summary, we demonstrated a noncontact method of patterning 2D materials with nanoscale precision at a very high processing speed. Our method preserves the material quality in unexposed areas and does not require protective layers that can contaminate the surface of the 2D materials. We patterned graphene monolayers, multilayers of MoS_2_, and PtSe_2_, suggesting that this approach is also applicable to a broad set of other 2D materials and thin films. Moreover, the XPS analysis of the patterned samples indicates no significant material degradation surrounding the exposure sites. As a direct writing method, this technique allows patterning arbitrary features in 2D during the same exposure. Combined with the direct growth and resist-free transfer of 2D materials and thin films, our method can expand the use of 2D materials in energy storage, optoelectronics, photonics, and sensing.

## Methods

### Sample Preparation

Glass coverslips (30 mm diameter, 170 μm ± 10 μm thick, double-side polished, borosilicate D263 glass, Thermo Fisher Scientific) were used as substrates, and the 2D material of choice was either deposited or grown on one surface of the coverslips. In the case of graphene, chemical vapor deposited graphene (Graphenea Inc., Spain) was transferred from copper foil to coverslips using a wet transfer technique. First, the monolayer graphene was spin-coated with poly(bisphenol A) carbonate (PC) (0.85 wt % in chloroform). Carbon residuals on the backside of the foil were removed in O_2_ plasma (power, 80 W; time, 120 s). The foil was placed onto the surface of an iron chloride (FeCl_3_) solution with the back side facing the liquid to release the graphene/PC stack from the growth substrate. The copper foil was etched entirely within 2 h, leaving the graphene/PC stack floating on the surface of the etching solution. Transferring the stack to deionized (DI) water first and then in a 9% HCl solution (15 min, respectively) removed residues of the etchant. After a final rinse in DI water for 15 min, the graphene/PC stack was transferred to the sample and dried on a hot plate at 45 °C for 15 min. Finally, submerging the sample in chloroform for several hours removed the covering PC layer.

Multilayer MoS_2_ film was grown by metalorganic chemical vapor deposition (MOCVD) at 800 °C and 2.5 mbar for 7 min (PlasmaPro 100 ICPCVD, Oxford Instruments) using H_2_S and Mo(CO)_6_ as precursors on a 200 mm diameter Si/SiO_2_ substrate, yielding a 3.5 nm thick uniform layer over the whole wafer. The 3.5 nm thickness corresponds to 5–6 monolayers of MoS_2_.^49,50^ The gas flows during the growth were as follows: 70 sccm He, 50 sccm Ar (carrier gas for Mo(CO)_6_), 6 sccm H_2_, and 5 sccm H_2_S

For transfer of the MoS_2_ films, the wafer was then cleaved into 5 × 5 mm^2^ samples. A PMMA layer was spin-coated on the MoS_2_ layer for support during wet transfer. The SiO_2_ was then etched in a 2 M KOH solution for 10 min. The delaminated PMMA/MoS_2_ layer floated at the solution’s surface and could be picked up by a glass coverslip. After double rinsed in DI water for 5 min, the floating PMMA/MoS_2_ was picked up with the final intended substrate (borosilicate coverslips). After transfer, the samples were desiccated for about 8 h. Then, the PMMA layer was stripped by immersion in acetone for 5 min and subsequently rinsed in IPA for 2 min. The acetone/IPA rinse was repeated once and followed by blow-drying with nitrogen.

Multilayer PtSe_2_ films were directly grown on top of the borosilicate coverslips. Initially, platinum (Pt) films of 1.4 and 2.8 nm thickness were sputtered onto the coverslips with a Cressington magnetron sputter coater. Synthesis of PtSe_2_ was achieved by converting these films using thermally assisted conversion (TAC), as discussed in previous publications.^41,45,46^ Selenium powder (>99.5% purity, VWR) was heated to 225 °C and transported toward the sample by a hydrogen flux of 100 sccm. The conversion occurred at a sample temperature of 450 °C, constant pressure of 10 mbar, and a process time of 120 min. All 2D material-coated coverslips were stored in normal environmental conditions.

### Sample Mounting and Focusing

A two-photon 3D printer (Photonic Professional GT2, Nanoscribe, Germany) was used to generate patterns in the 2D materials. The 2D materials were exposed in an oil immersion configuration using a 63× objective (Plan-Apochromat 63×/1.4 oil DIC M27, item no. 420782-9900-000, Zeiss, Germany) and applying a drop of immersion oil (Immersol 518F, Zeiss, Germany) on the coverslip surface without 2D material. The 2D material plane was then roughly focused by manually searching for the glass–air interface with the 2D material visible using the embedded microscope camera. At that stage, the built-in interface detection system of the two-photon 3D printer can find the glass–air interface with a resolution of less than 1 μm in the *z*-plane.

### Design Generation and Direct Writing

The printing job preparation program (Describe, Nanoscribe, Germany) was used to write, debug, and compile the code to generate the intended pattern design. In this study, we exposed individual spots to generate nanohole arrays, individual lines to outline device areas, and arrays of overlapping lines to remove the 2D material from the exposed area. Infrared femtosecond laser radiation (780 nm wavelength) was focused using the 63× objective through the glass substrate onto the 2D material. The laser power is equally split in a train of 80–120 fs long pulses with a repetition rate of 80 MHz. The maximum power which can be focused on the target using the 63× objective is around 40 mW, resulting in a maximum pulse energy of 500 pJ. The used laser pulse energy varied between 10 and 500 pJ, depending on the 2D material under exposure. A built-in laser scanning system was used to move the position of the laser beam, resulting in an effective speed for writing arrays of closed pack lines of up to 50 mm/s. In the case of nanohole arrays, individual spots were exposed for a variable amount of time, ranging from 100 ms down to 6 μs, which is the minimum exposure time allowed by the hardware. To facilitate the localization of the test patterns during characterization, we entirely removed the 2D material surrounding the test patterns by laser exposure (see Figure S1 in the Supporting Information).

### Scanning Electron Microscopy (SEM) of Line Width and Spot Size and Morphological Characterization of the Holes via Atomic Force Microscopy (AFM)

The patterned samples were imaged using scanning electron microscopy (Carl Zeiss AG-ULTRA 55, Carl Zeiss, Germany) without metallization and with low acceleration voltage (1 kV). The acquired images were analyzed using the ImageJ (NIH, USA) image analysis software. In the case of individual lines, the built-in measurement function (Analyze > Measure) was used to manually collect 20 measurements per line direction (vertical and horizontal) to account for beam aberration, resulting in 40 measurements per sample to estimate the line width of individual lines for a specific exposure parameter set. In the case of holes, the built-in particle analysis function (Analyze > Analyze Particles...) was used to analyze the acquired image after 8-bit conversion (Image > Type > 8-bit) and thresholding (Image > Adjust > Threshold... > Default) and to estimate the lateral dimension (diameter) of individual holes. The resulting outline mask was then manually compared with the original image to optimize the particle analysis parameters (size and circularity). The resulting area value was converted into a diameter and compared for different exposure parameter sets. When the image contrast did not allow for an accurate particle outlining, the same analysis for the individual lines was performed by manually measuring the diameters in horizontal and vertical directions.

AFM images were acquired using an Anasys NanoIR2 system atomic force microscope (Bruker, U.S.) using a tapping-mode tip PR-EX-T125-10 (Anasys) with a resonant frequency of ∼300 kHz. We used a scan size between 1 and 10 μm, a scan frequency between 0.5 and 1 Hz, and a resolution of at least 256 × 256 pixels.

### Raman Spectroscopy

All samples were characterized using an Alpha300 confocal Raman system (WITec, Germany) in ambient conditions. An excitation laser with a wavelength of 532 nm, a 100× NA = 0.9 objective, and an 1800 grooves/mm grating were used for all measurements. The excitation power was set to 1 mW for graphene, 0.5 mW for PtSe_2_, and 0.3 mW for MoS_2_. A motorized stage moved the sample with respect to the laser spot between position scans spanning a rectangular area (width between 8 and 200 μm). All Raman measurements were performed as area scans by recording consecutively multiple spectra (at least 25) at positions evenly distributed over the scanned area with integration times ranging from 4 to 8 s. We have included a table with the specific values used for scanning each material as Table S1 in the Supporting Information. The spectra depicted in the graphs are obtained by averaging over all spectra of an area scan. The extracted peak parameters used to generate the histograms depicted in Figure S6 of the Supporting Information were obtained by fitting Lorentzian peak shapes to the individual spectra of each area scan (25 spectra for PtSe_2_ and 50 for MoS_2_ and graphene). The fit parameters, such as peak position and fwhm, are then plotted as histograms.

### XPS Characterization

For the XPS characterization, we used a PHI VersaProbe III instrument (Physical Electronics, USA) equipped with a microfocused monochromated Al Kα source (1486.6 eV) and dual beam charge neutralization. Individual core orbitals of interest were characterized with high-resolution scans (specifically, molybdenum (Mo) and sulfur (S) were recorded for MoS_2_, platinum (Pt) and selenium (Se) for PtSe_2_). In addition, carbon (C), oxygen (O), and silicon (Si) were scanned in all samples. In the case of graphene, the carbon 1s orbital was recorded with lower pass energy, increased resolution, and integration time. The binding energy scale was referenced to the adventitious carbon 1s core level at 284.8 eV. The X-ray spot (100 μm in diameter) was focused in the center of a nanohole patterned 200 × 200 μm^2^ patch of the 2D material where the surrounding film has been entirely removed by the laser (see Figure S1 in the Supporting Information for the description of the geometry of test).

### Electrical Characterization

Metallic contact pads were deposited on the grown or transferred 2D materials by shadow evaporation before laser patterning. Specifically, a steel sheet (150 μm thick) was patterned by femtosecond laser ablation to obtain an array of rectangular openings (300 μm wide, 1 mm long, and with gaps of 100, 200, and 500 μm). The patterned steel sheet was glued on a squared metallic frame to avoid direct contact (roughly 300 μm gap) between the shadow mask and the 2D material and used as shadow mask for the subsequent metal evaporation. Then, a PAK 600 coating system (Provac, Germany) was used to deposit a titanium adhesion layer (at least 5 nm) and a gold contact layer (at least 50 nm) on the pristine 2D material. Then, the 2D material placed between the contact pads was patterned, generating nanohole arrays using the following parameters: 1 μm pitch between the center of each hole, 500 pJ pulse energy for graphene, 185 pJ for MoS_2_, and 90 pJ for PtSe_2_. In addition, the 2D material outside the active area of the devices was removed by laser ablation to electrically isolate the array of devices from the surrounding 2D material layer and define the channel geometry. For the electrical resistance measurement of the PtSe_2_ and MoS_2_ samples, a Rucker & Kolls 681A semiautomatic probe station with an Agilent E5270B Precision IV analyzer was used. Voltage sweeps in two-point configuration with currents kept below 100 μA were performed. The voltage range was adjusted according to the resistivity of each 2D material, specifically from −1 to 1 V in 10 mV steps for PtSe_2_ and from −20 to 20 V in 20 mV steps for MoS_2_. In the case of graphene, a Cascade Summit 11000-series manual probe station was used to measure the resistance of graphene channels in a four-point configuration with a linear current sweep starting from 100 μA to 300 μA with a step of 10 μA and without illuminating the sample under test.

## Supporting Information Available

AFM images of the laser patterned hole arrays of all three 2D materials, ablation regimes in the different materials, replotting of Figure 2b using Liu-type plots, processing speed, schematic illustration of laser patterned structures for Raman and XPS analyses, recording of Raman area scans, Raman characterization of the pristine 2D materials on glass coverslips, high-resolution Raman imaging of patterned and pristine 2D material films, comparison between pristine and laser structured 2D materials by XPS, XPS analysis of cleared PtSe_2_ and MoS_2_ areas, and electrical characterization of pristine and patterned 2D materials (PDF)
