# Supplementary material for: Ultrafast and Resist-Free Nanopatterning of 2D Materials by Femtosecond Laser Irradiation
Source: ACS Nano. 2023 Apr 19;17(9):8041–52. doi: 10.1021/acsnano.2c09501 (PMC10173691; doi:10.1021/acsnano.2c09501)
Supplement: Supplementary file 1 — nn2c09501_si_001.pdf [file nn2c09501_si_001.pdf]

Supporting Information

Ultrafast and resist-free nanopatterning of 2D materials by femtosecond laser irradiation

Alessandro Enrico‡, Oliver Hartwig‡, Nikolas Dominik, Arne Quellmalz, Kristinn B. Gylfason, Georg S. Duesberg, Frank Niklaus*, and Göran Stemme**

Alessandro Enrico, Arne Quellmalz, Kristinn B Gylfason, Frank Niklaus, Göran Stemme

Division of Micro and Nanosystems, KTH Royal Institute of Technology, Malvinas väg 10, 10044, Stockholm, Sweden

*Email: frank@kth.se, stemme@kth.se

Oliver Hartwig, Nikolas Dominik, Georg S Duesberg

Institute of Physics, EIT 2, Faculty of Electrical Engineering and Information Technology, University of the Bundeswehr Munich & SENS Research Center, Werner-Heisenberg-Weg 39, 85577, Neubiberg, Germany

*Email: duesberg@unibw.de

‡These authors contributed equally.

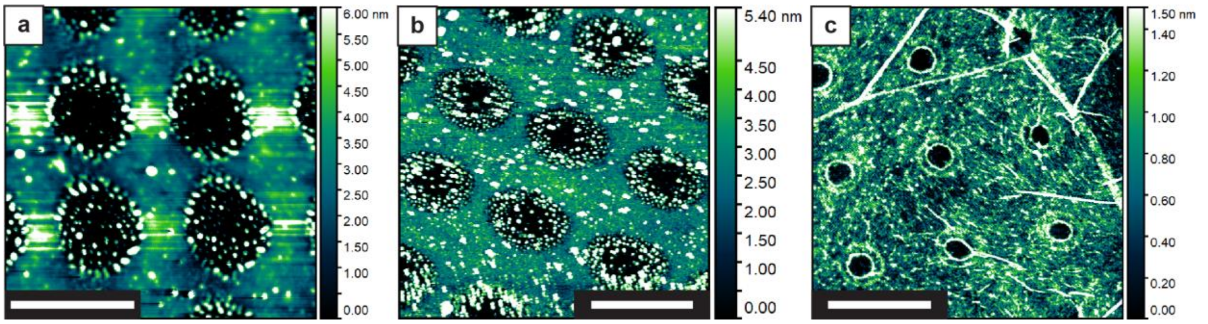

Figure S1– AFM images of the laser patterned hole arrays of all three 2D materials. a) Holes in $PtSe_2$ film. Islands mainly composed of platinum can be found at the edge of and inside the illuminated area. b) Holes in MoS_2 film. Clusters of MoO_x can be found at the edges and inside the illuminated area. c) Holes in graphene. In contrast with $PtSe_2$ and MoS_2, no island formation is observed around or inside the patterned area for the graphene. Scale bars, 1 µm.

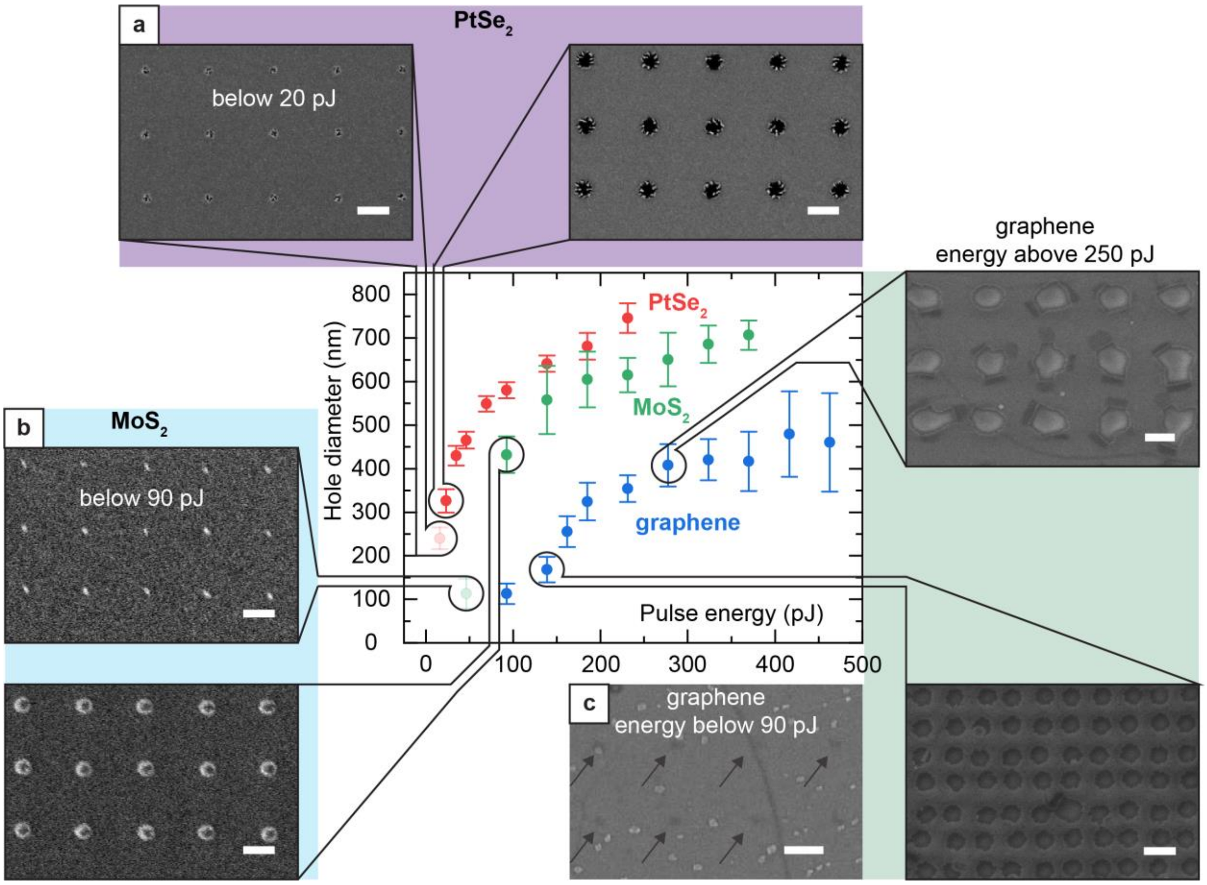

Figure S2 – Ablation regimes in the different materials. The graph in the center is identical to Figure 2c with the addition of two data points for $PtSe_2$ and MoS_2 (semitransparent ones on the left) corresponding to illuminated positions that feature modifications but not complete holes. a) and b) compare the difference between the laser exposure results on $PtSe_2$ and MoS_2, respectively, when using pulse energy above and slightly below the threshold for hole formation. c) For a graphene monolayer, pulse energies below 90 pJ can only produce a lifting of the layer or a material change (defects and oxidation) but no hole. Increasing the pulse energy produces circular holes. At higher pulse energy, folding of the graphene monolayer produces straight edges in the hole profile. Scale bars, 500 nm for all pictures.

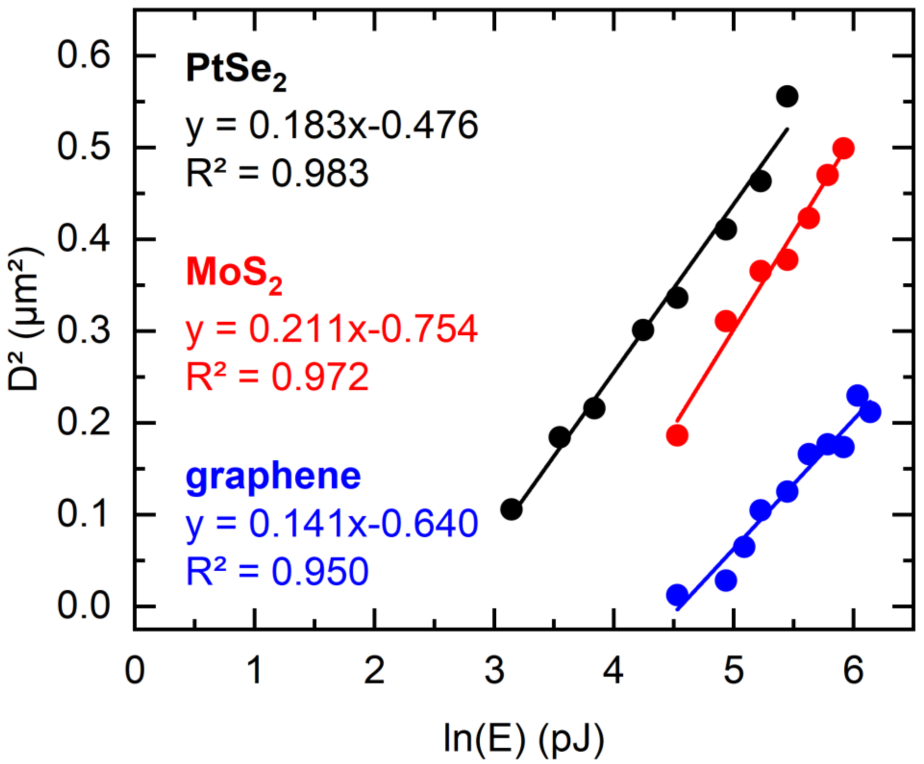

Figure S3 - Replotting of Figure 2c using Liu-type plots. The angular coefficients do not match despite using the same magnifying objective. We attribute the deviation from the expected dependence to a combination of multipulse exposure (800 pulses), the predominant removal of one of the two species composing the dichalcogenides 2D materials, and the flapping/folding of graphene at high pulse energies.

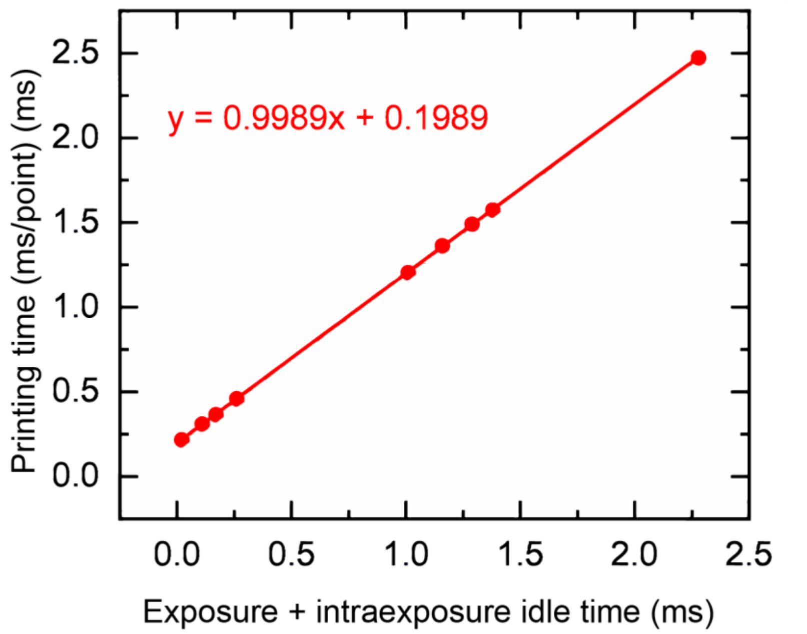

Figure S4 – Processing speed. We performed a simulation through the Describe software and an experiment measuring the processing time for large nanohole arrays (at least 600 x 600 elements matrix). The minimum exposure setting on the tool for which we observed an exposure was 6 µs. We characterized the idle time, mainly due to the mirror moving the beam to the following position, to be around 200 µs.

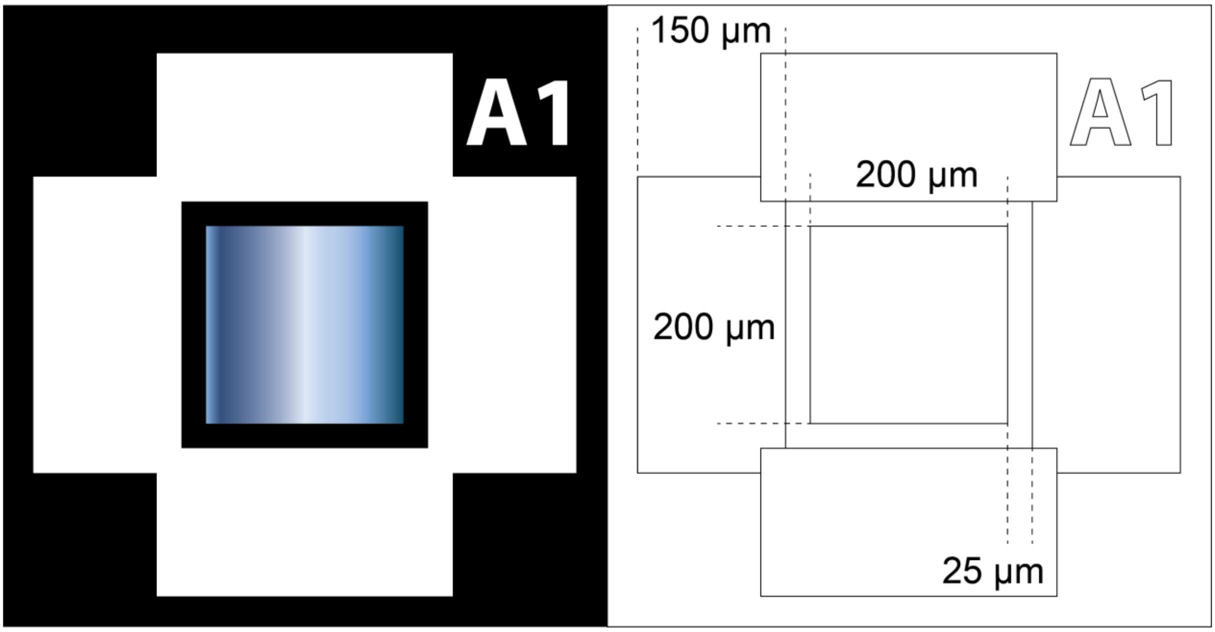

Figure S5 – Schematic illustration of laser patterned structures for Raman and XPS analyses. Black, white, and color gradient in the left image refer to areas with pristine 2D materials, areas where the 2D material was entirely removed, and patterned 2D materials, respectively. The sketch on the right reports the dimensions of the different areas. These structures were then analyzed by Raman spectroscopy and XPS. Specifically, for the XPS measurements, the evaluated area was the central square area (200 µm x 200 µm) with the patterned nanohole array. The size of the area was selected to match the spot size of the X-ray beam (100 µm diameter) of the used XPS system. In these areas, the laser was either intermittently shone to pattern a nanohole array or an array of individual lines. The area surrounding the test structures was exposed to completely remove the 2D material, creating a 150 µm wide frame that provides optical and XPS contrasts to facilitate the localization of the test structure on the substrate. In addition, laser direct writing was also used to label each test structure and link it to a specific pulse energy-pitch combination.

Recording of Raman area scans:

To assess the initial quality of the grown or transferred 2D materials on the glass coverslips, we characterized the layers by Raman spectroscopy (**Figure S6**, details in the Method section in and in **Table S1**). The Raman spectrum of the pristine $PtSe_2$ film features the E_g mode peak at 178.8 cm^{-1} and the A_{1g} mode peak at 206.9 cm^{-1}, which agree with the values presented in the Results and Discussion section and with previously reported values in the literature (**Figure S6a**).[1–5]

While the intensities of the E_g and A_{1g} modes in bulk $PtSe_2$ are normally of similar magnitude, the intensity of the observed A_{1g} mode of the grown $PtSe_2$ films was three times

Table S1: Parameters used for the Raman area scans (results in Figure S6).

Material	$PtSe_2$	MoS_2	graphene
n (points in horizontal direction)	5	10	10
m (points in vertical direction)	5	5	5
width w (µm)	30	200	149
height h (µm)	30	100	85
integration time (s)	6	6	8
laser power (mW)	0.5	0.2	1

lower than the E_g mode intensity, confirming the few-layer nature of the $PtSe_2$ film.[1] Moreover, the average full-width at half-maximum (FWHM) value was around 5.5 cm^{-1}, indicating high crystallinity of the grown $PtSe_2$ film (**Figure S6b**).[3,4] The low standard deviation of the width of the E_g mode (0.15 cm^{-1}) suggested a high homogeneity of the film.[4]

In the case of MoS_2, the Raman spectrum of the transferred films featured the E^1_{2g} mode peak at 383.7 cm^{-1} and the A_{1g} mode peak at 407.7 cm^{-1}, which is consistent with previously reported values (**Figure S6c**).[6–9] The mode difference A_{1g} - E^1_{2g} is centered around 24.2 cm^{-1} confirming the few-layer nature of the MoS_2 (**Figure S6d**).[9,10] Similar to $PtSe_2$, the low standard deviation value (0.28 cm^{-1}) indicates a high uniformity of the layer thickness.[11,12]

The characteristic modes (2D and G bands) for graphene are observed at 2686 cm^{-1} and 1595 cm^{-1}, which is in line with previous reports (**Figure S6e**).[11,13–16] The intensity of the defect-activated D band (located at 1349 cm^{-1}) was significantly lower than the intensity of the G band ($I_D/I_G < 0.1$), suggesting a high quality of the transferred graphene.[16] The large peak intensity ratio (I_{2D}/I_G) of approximately 4 confirmed the monolayer nature of the graphene

film.[14] The narrow FWHM of the 2D band averaged to 33 cm^{-1}, which is comparable to reported values for monolayer graphene (**Figure S6f**).[11,15]

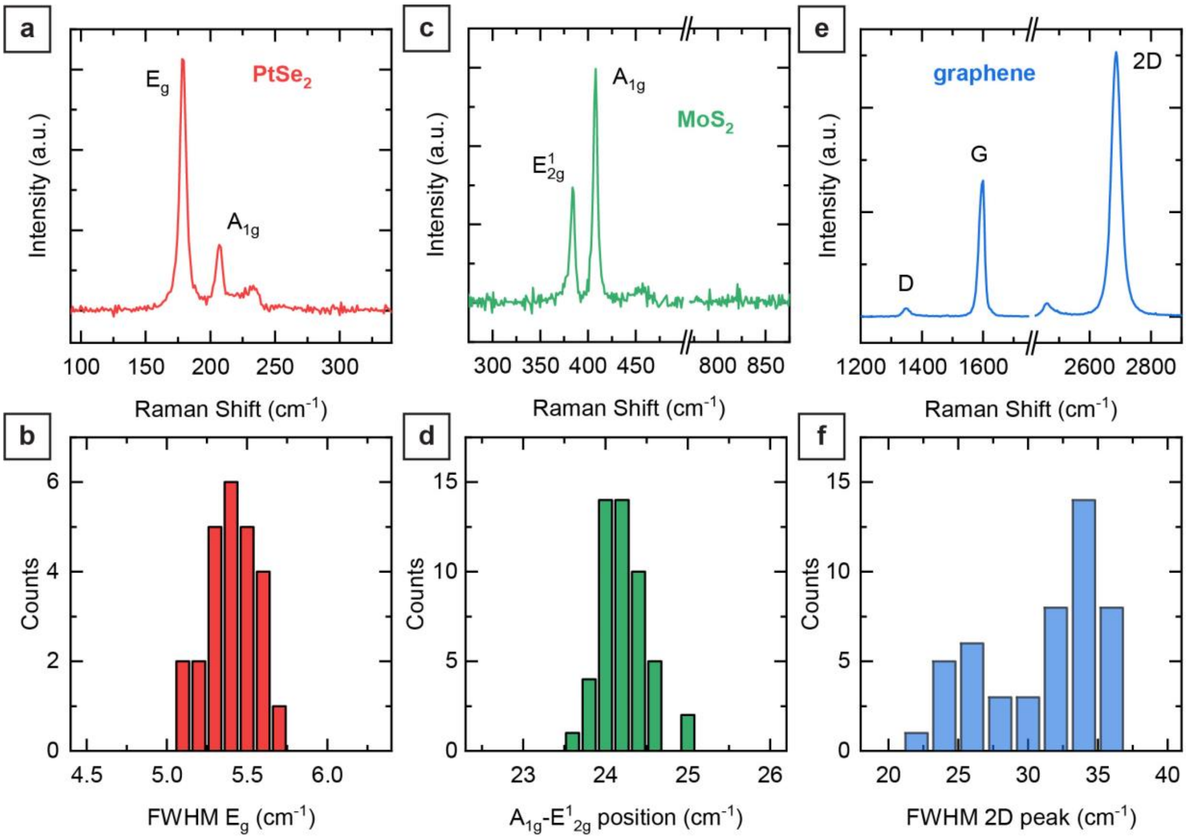

Figure S6 – Raman characterization of the pristine 2D materials on glass coverslips. a) Raman spectrum of the $PtSe_2$ film grown directly onto the glass coverslip. b) Full-width at half-maximum (FWHM) of the E_g mode of $PtSe_2$, indicating high film crystallinity. c) Raman spectrum of the MoS_2 film transferred onto the coverslip. d) Mode difference A_{1g}–E^1_{2g} for MoS_2, confirming the multilayer nature of the film. e) Raman spectrum of graphene, showing a small defect-activated band D. f) FWHM of the 2D mode of graphene, confirming the monolayer nature of the film. The spectra in a), c), and e) are the results of averaging all measurement positions obtained by Raman mapping. To generate the histograms in b), d), and f), we recorded the spectra of individual positions using a laser spot size of ~ 500 nm and an integration time of at least 6 s, then moved the sample by at least 6 µm using a motorized stage and recorded the spectrum of that region. This process was repeated to scan a square matrix of at least 25 elements on a defined area. We have included a table with the specific values used for each material as Table S1 in the Supporting Information. The peak parameters used to generate the histograms were obtained by fitting the individual spectra of each area scan using Lorentzian peak shapes (25 fitted spectra for $PtSe_2$, 50 for MoS_2 and graphene). The extracted fit parameters, such as peak position and FWHM, are then plotted as histograms. The data from the multiple positions were then averaged to get a representative spectrum for each material.

High-resolution Raman imaging:

To analyze the uniformity and conditions of the unexposed areas of 2D materials near the holes, we repeated the Raman characterization using a distance of 300 nm between each measurement on patterned and pristine areas to obtain the highest resolution of mapping possible with the spot size of the laser beam used in the Raman characterization setup. For this characterization, we patterned holes with 1 µm and high pulse energy (500 pJ). We used the highest intensity mode for each material to generate a heat map and compare those areas with corresponding pristine films. We get comparable signal intensities for all materials from pristine films and regions surrounding the illuminated spots. We only observe a weaker signal for patterned $PtSe_2$ films since the dimensions of the holes were larger than the ones in patterned MoS_2 and graphene layer analyzed here. The remaining undamaged $PtSe_2$ regions between the holes are smaller than the laser spot size and, thus, result in a lower signal intensity. The Raman scans were performed on 4x4 $µm^2$ areas with 0.33 µm steps between the measurement positions (12 x 12 spectra). We get comparable signal intensities for all materials from pristine films and regions surrounding the illuminated spots. We only observe a weaker signal for patterned $PtSe_2$ films since the dimensions of the holes were larger than the ones in patterned MoS_2 and graphene layer analyzed here. The remaining undamaged $PtSe_2$ regions between the holes are smaller than the laser spot size and, thus, result in a lower signal intensity.

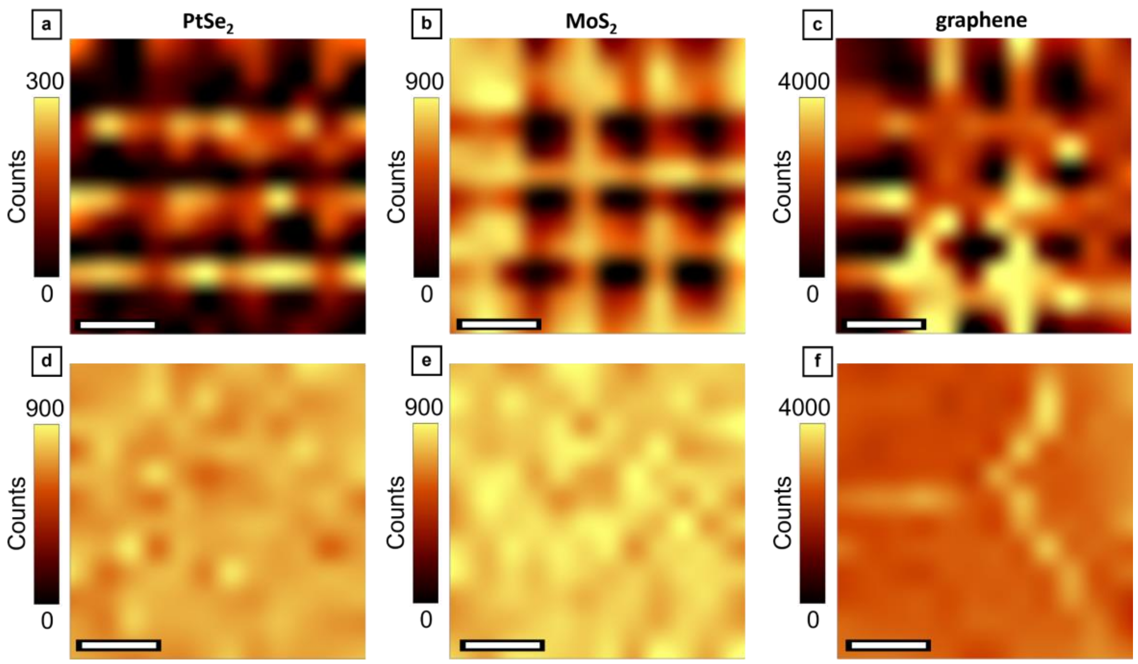

Figure S7 – High-resolution Raman imaging of patterned and pristine 2D material films. a), b), and c) are Raman maps of patterned $PtSe_2$, MoS_2, and graphene films, respectively. d), e), and f) are Raman maps of pristine $PtSe_2$, MoS_2, and graphene films, respectively. The heat map results from the integrated intensity of the highest mode in each of the 2D materials: E_g mode for $PtSe_2$, A_{1g} mode of the MoS_2, and 2D mode for graphene. Scale bar, 1 µm for all maps.

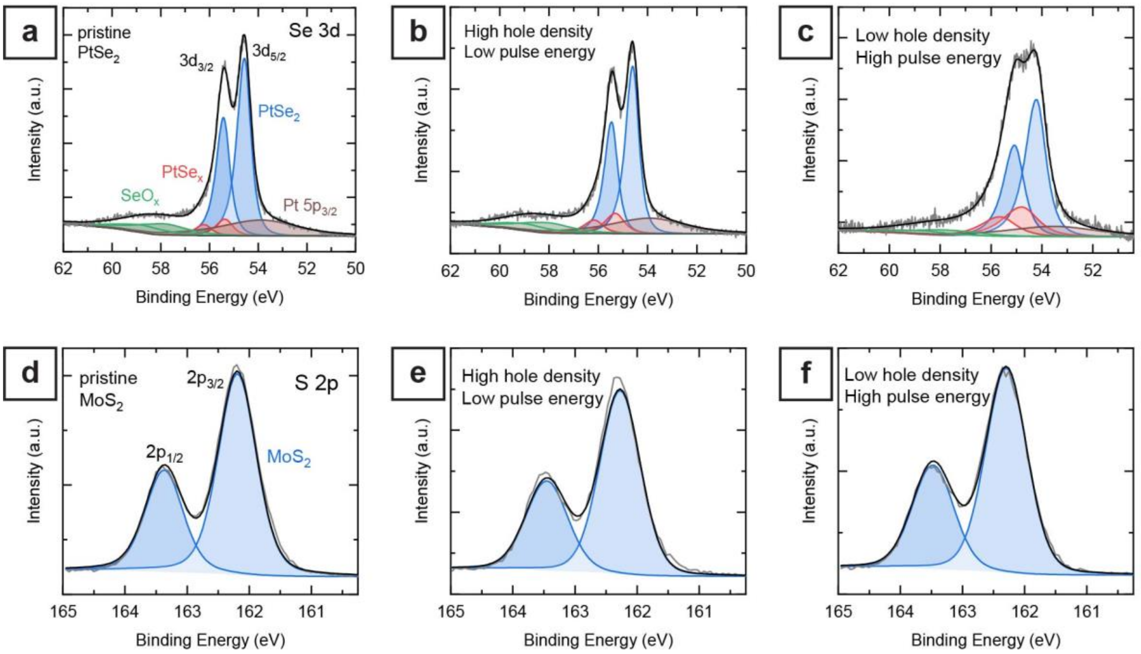

Figure S8 – Comparison between pristine and laser structured 2D materials by XPS. a) Characteristic Se 3d spectrum of pristine $PtSe_2$ with the Se 3d doublet binding energies at 54.6 eV for $3d_{5/2}$ and 55.5 eV for $3d_{3/2}$. b) Se 3d orbital of $PtSe_2$ film exposed using a high-density nanohole pattern (300 nm hole-to-hole pitch) and using low pulse energy (20 pJ). c) Se 3d orbital of $PtSe_2$ film exposed using a low-density nanohole array (1 µm hole-to-hole pitch) and high pulse energy (500 pJ). d) Characteristic S 2p spectrum of the transferred MoS_2 film with the S 2p doublet binding energies at 162.2 eV for $2p_{3/2}$ and 163.4 eV for $2p_{1/2}$. e) S 2p orbital of MoS_2 film exposed using a high-density nanohole pattern (300 nm hole-to-hole pitch) and using low pulse energy (100 pJ). f) S 2p orbital of MoS_2 film exposed using a low-density nanohole array (4 µm hole-to-hole pitch) and high pulse energy (500 pJ).

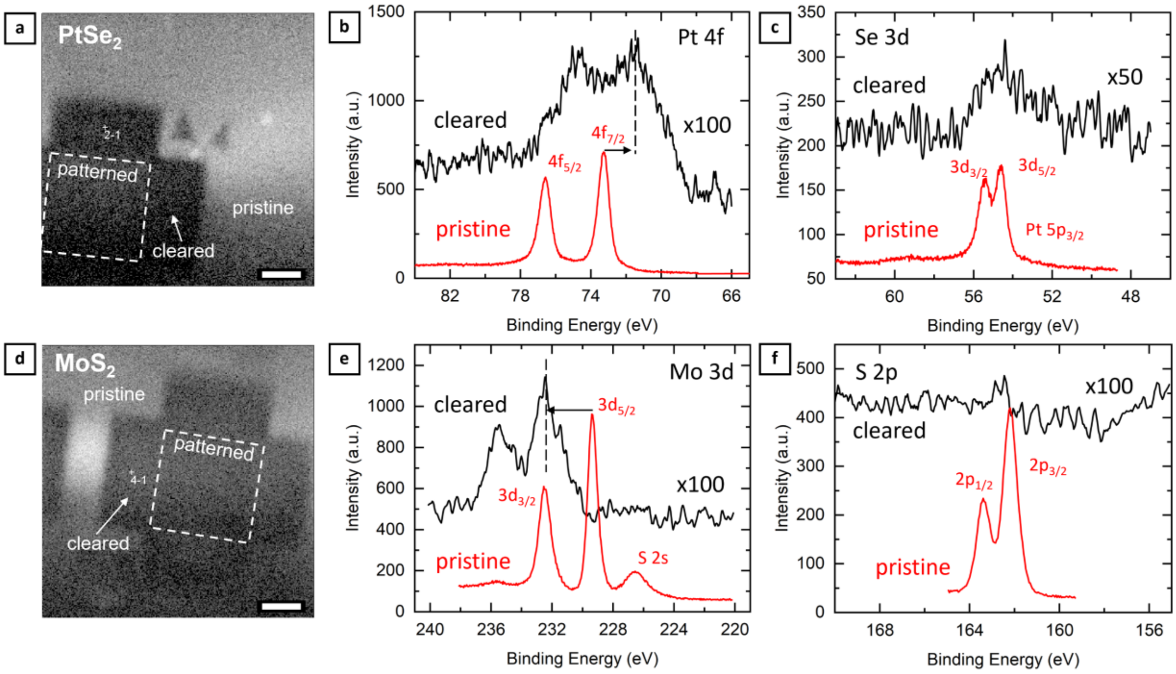

Figure S9 – XPS analysis of cleared $PtSe_2$ and MoS_2 areas. a) Scanning X-ray-induced secondary electron image (SXI) of the laser patterned geometry in a $PtSe_2$ film. The XPS spectra were recorded on pristine and cleared areas. b) Recorded Pt 4f orbitals of cleared and pristine $PtSe_2$ areas. Lower intensity and peak shift to lower binding energy are observed for the cleared area spectrum. c) Recorded Se 3d orbitals. Only traces of Se are observed, presumably bound to the Pt atoms in a substoichiometric form of $PtSe_x$. d) SXI image of the laser patterned geometry in a MoS_2 film. e) Recorded Mo 3d orbitals. The signal of the cleared area shows low-intensity modes of oxidized Mo as MoO_x. f) Recorded S 2p orbitals. No sulfur signal is observed for the cleared areas, concluding that no MoS_2 remained in the cleared areas. Scale bar, 100 µm for both images. In b, c), e, and f), the spectra of the cleared area have low intensities and are presented with different scaling factors from 1x for better visibility: 100x in b), e) and f), and 50x in c).

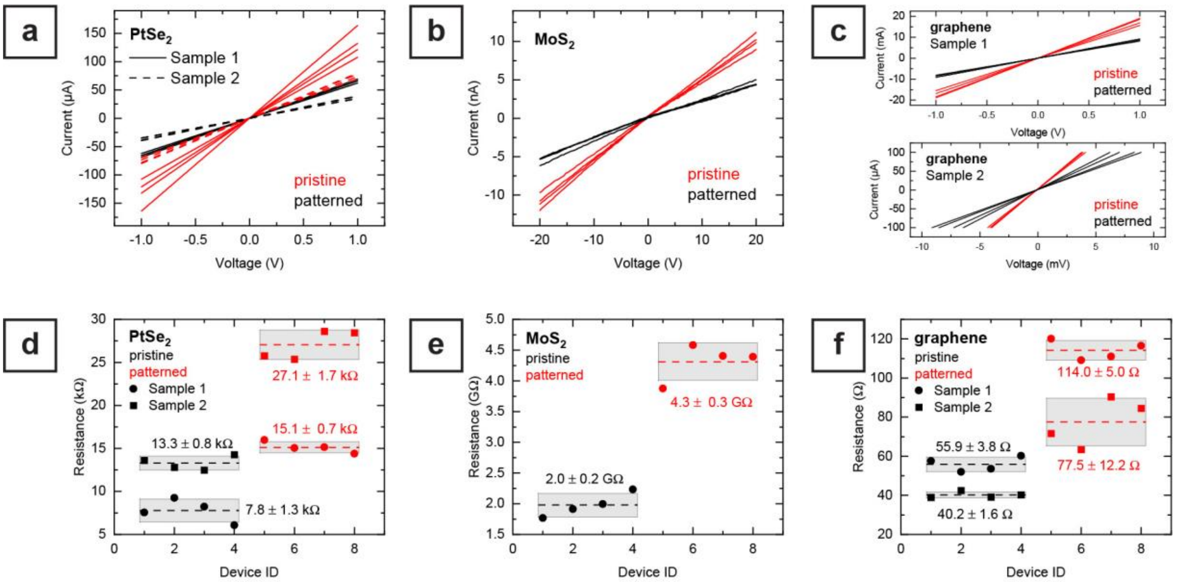

Figure S10– Electrical characterization of pristine and patterned 2D materials. a-c) Current-voltage characteristics of pristine and patterned a) $PtSe_2$, b) MoS_2, and c) graphene channels. d-f) Resistance values of each pristine and patterned 2D materials channel. The dashed lines indicate the average resistance, and the shaded regions indicate the standard deviation. Variation in the average resistance is observed among both $PtSe_2$ and graphene samples. For TAC-synthesized $PtSe_2$, resistance deviations in films of similar thickness were previously observed for the growth of thin films (<8 nm).[4] For graphene and MoS_2 films, deviations in the CVD growth and the strain caused by the wet-transfer are likely the cause of fluctuations in the film resistivity.[11,17]

SUPPORTING INFORMATION REFERENCES

(1) O'Brien, M.; McEvoy, N.; Motta, C.; Zheng, J.-Y.; Berner, N. C.; Kotakoski, J.; Elibol, K.; Pennycook, T. J.; Meyer, J. C.; Yim, C.; Abid, M.; Hallam, T.; Donegan, J. F.; Sanvito, S.; Duesberg, G. S. Raman Characterization of Platinum Diselenide Thin Films. *2D Mater.* **2016**, *3* (2), 021004. https://doi.org/10.1088/2053-1583/3/2/021004.

(2) Yim, C.; Lee, K.; McEvoy, N.; O'Brien, M.; Riazimehr, S.; Berner, N. C.; Cullen, C. P.; Kotakoski, J.; Meyer, J. C.; Lemme, M. C.; Duesberg, G. S. High-Performance Hybrid Electronic Devices from Layered PtSe 2 Films Grown at Low Temperature. *ACS Nano* **2016**, *10* (10), 9550–9558. https://doi.org/10.1021/acsnano.6b04898.

(3) Szydłowska, B. M.; Hartwig, O.; Tywoniuk, B.; Hartman, T.; Stimpel-Lindner, T.; Sofer, Z.; McEvoy, N.; Duesberg, G. S.; Backes, C. Spectroscopic Thickness and Quality Metrics for PtSe2 Layers Produced by Top-down and Bottom-up Techniques. *2D Mater.* **2020**, *7* (4). https://doi.org/10.1088/2053-1583/aba9a0.

(4) Lukas, S.; Hartwig, O.; Prechtl, M.; Capraro, G.; Bolten, J.; Meledin, A.; Mayer, J.; Neumaier, D.; Kataria, S.; Duesberg, G. S.; Lemme, M. C. Correlating Nanocrystalline Structure with Electronic Properties in 2D Platinum Diselenide. *Adv. Funct. Mater.* **2021**, *31* (35), 2102929. https://doi.org/10.1002/adfm.202102929.

(5) Prechtl, M.; Parhizkar, S.; Hartwig, O.; Lee, K.; Biba, J.; Stimpel-Lindner, T.; Gity, F.; Schels, A.; Bolten, J.; Suckow, S.; Giesecke, A. L.; Lemme, M. C.; Duesberg, G. S. Hybrid Devices by Selective and Conformal Deposition of PtSe2 at Low Temperatures. *Adv. Funct. Mater.* **2021**. https://doi.org/10.1002/adfm.202103936.

(6) Wieting, T. J.; Verble, J. L. Infrared and Raman Studies of Long-Wavelength Optical Phonons in Hexagonal MoS2. *Phys. Rev. B* **1971**, *3* (12), 4286–4292. https://doi.org/10.1103/PhysRevB.3.4286.

(7) Splendiani, A.; Sun, L.; Zhang, Y.; Li, T.; Kim, J.; Chim, C. Y.; Galli, G.; Wang, F. Emerging Photoluminescence in Monolayer MoS2. *Nano Lett.* **2010**, *10* (4), 1271–1275. https://doi.org/10.1021/nl903868w.

(8) Gatensby, R.; McEvoy, N.; Lee, K.; Hallam, T.; Berner, N. C.; Rezvani, E.; Winters, S.; O'Brien, M.; Duesberg, G. S. Controlled Synthesis of Transition Metal Dichalcogenide Thin Films for Electronic Applications. *Appl. Surf. Sci.* **2014**, *297*, 139–146. https://doi.org/10.1016/j.apsusc.2014.01.103.

(9) Li, H.; Zhang, Q.; Yap, C. C. R.; Tay, B. K.; Edwin, T. H. T.; Olivier, A.; Baillargeat, D. From Bulk to Monolayer MoS 2: Evolution of Raman Scattering. *Adv. Funct. Mater.* **2012**, *22* (7), 1385–1390. https://doi.org/10.1002/adfm.201102111.

(10) Hartwig, O.; Duesberg, G. S. Raman Spectroscopy of Stacked 2D-Materials. *Imaging Microsc.* **2020**.

(11) Quellmalz, A.; Wang, X.; Sawallich, S.; Uzlu, B.; Otto, M.; Wagner, S.; Wang, Z.; Prechtl, M.; Hartwig, O.; Luo, S.; Duesberg, G. S.; Lemme, M. C.; Gylfason, K. B.; Roxhed, N.; Stemme, G.; Niklaus, F. Large-Area Integration of Two-Dimensional Materials and Their Heterostructures by Wafer Bonding. *Nat. Commun.* **2021**, *12* (1), 1–11. https://doi.org/10.1038/s41467-021-21136-0.

(12) Luo, S.; Cullen, C. P.; Guo, G.; Zhong, J.; Duesberg, G. S. Investigation of Growth-Induced Strain in Monolayer MoS2 Grown by Chemical Vapor Deposition. *Appl. Surf. Sci.* **2020**, *508*, 145126. https://doi.org/10.1016/j.apsusc.2019.145126.

(13) Siokou, A.; Ravani, F.; Karakalos, S.; Frank, O.; Kalbac, M.; Galiotis, C. Surface Refinement and Electronic Properties of Graphene Layers Grown on Copper Substrate: An XPS, UPS and EELS Study. *Appl. Surf. Sci.* **2011**, *257* (23), 9785–9790. https://doi.org/10.1016/j.apsusc.2011.06.017.

(14) Yim, C.; McEvoy, N.; Duesberg, G. S. Characterization of Graphene-Silicon Schottky Barrier Diodes Using Impedance Spectroscopy. *Appl. Phys. Lett.* **2013**, *103* (19). https://doi.org/10.1063/1.4829140.

(15) Kumar, S.; McEvoy, N.; Lutz, T.; Keeley, G.; Whiteside, N.; Blau, W.; Duesberg, G. S. Low Temperature Graphene Growth. *ECS Trans.* **2009**, *19* (5), 175–181. https://doi.org/10.1149/1.3119541.

(16) Dresselhaus, M. S.; Jorio, A.; Souza Filho, A. G.; Saito, R. Defect Characterization in Graphene and Carbon Nanotubes Using Raman Spectroscopy. *Philos. Trans. R. Soc. A Math. Phys. Eng. Sci.* **2010**, *368* (1932), 5355–5377. https://doi.org/10.1098/rsta.2010.0213.

(17) Hallam, T.; Berner, N. C.; Yim, C.; Duesberg, G. S. Strain, Bubbles, Dirt, and Folds: A Study of Graphene Polymer-Assisted Transfer. *Adv. Mater. Interfaces* **2014**, *1* (6), 1–7. https://doi.org/10.1002/admi.201400115.
